# Dual Functional Dopant‐Free Contacts with Titanium Protecting Layer: Boosting Stability while Balancing Electron Transport and Recombination Losses

**DOI:** 10.1002/advs.202202240

**Published:** 2022-06-15

**Authors:** Zhaolang Liu, Hao Lin, Zilei Wang, Liyan Chen, Taojian Wu, Yicong Pang, Lun Cai, Jian He, Shanglong Peng, Hui Shen, Pingqi Gao

**Affiliations:** ^1^ School of Materials Sun Yat‐sen University Guangzhou 510275 P. R. China; ^2^ National and Local Joint Engineering Laboratory for Optical Conversion Materials and Technology School of Materials and Energy Lanzhou University Lanzhou 730000 P. R. China; ^3^ Institute for Solar Energy Systems Guangdong Provincial Key Laboratory of Photovoltaic Technology State Key Laboratory of Optoelectronic Materials and Technologies Sun Yat‐sen University Guangzhou 510275 P. R. China; ^4^ Jiangsu Collaborative Innovation Center of Photovoltaic Science and Engineering Changzhou University Changzhou 213164 P. R. China

**Keywords:** carrier‐selective contacts, dopant‐free, electron transport, heterojunction solar cells, passivating contacts

## Abstract

Combining electron‐ and hole‐selective materials in one crystalline silicon (Si) solar cell, thereby avoiding any dopants, is not considered for application to photovoltaic industry until only comparable efficiency and stable performance are achievable. Here, it is demonstrated how a conventionally unstable electron‐selective contact (ESC) is optimized with huge boost in stability as well as improved electron transport. With the introduction of a Ti thin film between a‐Si:H(*i*)/LiF and Al electrode, high‐level passivation (*S*_eff_ = 4.6 cm s^–1^) from a‐Si:H(*i*) and preferential band alignment (*ρ*_C_ = 7.9 mΩ cm^2^) from low work function stack of LiF/Ti/Al are both stably retained in the newly constructed *n*‐Si/a‐Si:H(*i*)/LiF/Ti/Al ESC. A detailed interfacial elements analysis reveals that the efficiently blocked inward diffusion of Al from electrode by the Ti protecting layer balances transport and recombination losses in general. This excellent electron‐selective properties in combination with large process tolerance that enable remarkable device performance, particularly high efficiencies of 22.12% and 23.61%, respectively, are successfully approached by heterojunction solar cells with dopant‐free ESC and dopant‐free contacts for both polarities.

## Graphical abstract

A universal strategy is presented to block the inward diffusion of Al from the electrode into the a‐Si:H(*i*) layer using thermally evaporated Ti as a protecting layer, with the preferred structure of n‐Si/a‐Si:H(*i*)/LiF/Ti/Al achieving excellent thermal stability of the passivation and contact properties. Furthermore, the silicon heterojunction solar cells with interdigitated back dopant‐free contacts possess a record efficiency of 23.61%.

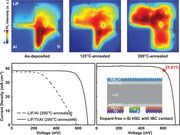

## 1 Introduction

Elevating the competitiveness of photovoltaic (PV) devices in the renewable energy community relies on the ongoing technology innovations to promote the module efficiency while reducing the manufacturing cost. Generally, the improvement of device efficiency can be realized by integrating advanced structural designs and/or new material systems coupled with suitable processing solutions. With the implementation of these progresses, various types of solar cells (SCs), including crystalline silicon (c‐Si) SCs, perovskite SCs and perovskite/c‐Si tandem SCs, etc.,^[^ ^1^ ^]^ keep making new breakthroughs in efficiency and some of them are approaching theoretical Shockley–Queisser limits.^[^ ^2^, ^3^, ^4^ ^]^ The simplification of processes and the diversification of material choices, which of course are favorable to both efficiency and cost, pose however new challenges to device stability. The unsatisfied efficiency and performance degradation of PV devices that is particularly relevant for the cases of functional materials or/and carrier contacts underlying technological process or high‐humidity environment, can be traced back to the denaturation of the related materials, interfacial reaction, ion diffusion as well as heat loss, etc.^[^ ^5^, ^6^, ^7^ ^]^ This sets up an obstacle for approaching high‐efficiency and consequently inhibiting device commercialization.

In comparison with the device structure with direct contact of c‐Si and metal electrodes, the construction of heterojunction SCs (HSCs) by introducing functional materials as passivating layer (to reduce the density of interface defects) and carrier transport layer (to selectively conduct only one polarity of charges), respectively, can effectively eliminate the adverse impact of heavy carrier recombination in contact regions, achieving higher open‐circuit voltages (*V*_OC_s) and power conversion efficiencies (PCEs).^[^ ^8^, ^9^, ^10^ ^]^ For example, heterointrinsic thin layer (HIT) SCs exhibit ultrahigh device performance by leveraging the excellent passivation of hydrogenated intrinsic amorphous silicon [a‐Si:H(*i*)] and introducing doped a‐Si:H to form carrier‐selective passivation contacts for both polarities.^[^ ^9^, ^11^ ^]^ Similar design is also found for the structure of SiO*_x_*/poly‐Si in tunnel oxide passivated contact (TOPCon) SCs.^[^ ^10^, ^12^ ^]^ In addition, replacing the doped electron/hole transport layers (i.e., doped a‐Si:H in HIT and doped poly‐Si in TOPCon) with low/high work function materials such as alkali and alkaline earth metals,^[^ ^13^, ^14^ ^]^ their oxides and fluorides,^[^ ^15^, ^16^, ^17^, ^18^ ^]^ and transition metal oxides (TMO)^[^ ^19^, ^20^, ^21^ ^]^ to form dopant‐free passivating contacts are also a promising approach to reduce parasitic absorption and lower fabrication cost.^[^ ^22^, ^23^, ^24^ ^]^ However, potential instability also goes along with the presence of additional materials and interfaces in this type of HSCs, such as the deterioration of passivation property of a‐Si:H(*i*) at elevated processing temperatures, the degradation of carrier‐selective properties of high work function TMO films owing to the oxygen vacancies created by process, and reactions caused by the metal atoms diffusion from electrodes.^[^ ^25^, ^26^ ^]^ A wide range of material options for heterojunction devices screens more stable materials as transport layers. However, as an indispensable process in the fabrication of solar cells, the deleterious consequence of metallization on device performance must be well addressed. Especially for a‐Si:H(*i*) films, as an unstable system, it tends to spontaneously undergo atomic rearrangement to reach a lower free energy state. When a‐Si:H(*i*) comes in contact with metal films, particularly Al, Al‐Si mixed‐phases with lower binding energy, or even aluminum silicide (Al*_X_* Si) and Al‐doped a‐Si:H(*p*) layer, will form at the interface at low temperatures (150–200 °C) due to their high diffusion coefficients within each other.^[^ ^27^, ^28^, ^29^ ^]^ On this basis, metal‐induced crystallization will occur at higher temperatures (200–300 °C) as the Si atomic rearrangement receives an additional driving force from the metallic film.^[^ ^30^, ^31^ ^]^ All these changes in the a‐Si:H(*i*) layer cause a dramatic deterioration of the associated properties, including passivation and carrier transport properties, leading in the degradation of device performance.

Few strategies have been reported for the problems caused by the metal ion diffusion in HSCs, where a viable one is the insertion of an isolation layer between the metal and the corresponding functional layer to avoid direct contact. The transparent conducting oxide (TCO) layers in HIT SCs are a successful example that ensures sufficiently lateral conductivity and separation of the metal electrodes from the doped a‐Si:H. However, for future‐oriented PV techniques, TCO layers are no longer a good choice due to their higher parasitic absorption as well as economic considerations.^[^ ^32^ ^]^ On the other hand, the atomic‐layer‐deposition (ALD)‐grown films (TiO*_X_*,^[^ ^14^, ^33^, ^34^, ^35^ ^]^ TiN*_X_*,^[^ ^36^ ^]^ TiO*_X_* N*_Y_*,^[^ ^37^ ^]^ TaN*_X_*,^[^ ^38^ ^]^ ZnO*_X_*,^[^ ^39^, ^40^ ^]^ etc.) have a high density, allowing them to be employed as protecting layers in innovative electron‐selective contact (ESC) designs. A large amount of these ESC designs can ensure Ohmic contact with a low initial contact resistivity (*ρ*_C_) value (<10 mΩ cm^2^) at room temperature, however, usually fail to withstand annealing treatment at 200 °C (a minimum threshold temperature for processing c‐Si based SCs). Especially for the case of a‐Si:H(*i*) as passivation layer, this threshold temperature was further reduced to below 150 °C. The introduction of ALD‐grown thin layer of metal oxide/nitride between the low work function layer and the a‐Si:H(*i*) is reported with enhanced stability of the ESC designs. However, this approach has encountered technical difficult in balancing the passivation characteristics and the contact resistance, showing increased initial *ρ*_C_ of the ESC by more than one order of magnitude due to the poor intrinsically electrical conductivity inherent to the dielectric protecting layer, as shown in Figure S1 in the Supporting Information. Therefore, as a candidate material of the protecting layer, it should have good compactness and electrical conductivity to suppress the harmful effects from some fabrication processes with elevated temperature as well as the sequential metallization process without sacrificing the selectivity of ESCs on transporting electrons.

As we all know, Ti thin film has been successfully used as an adhesive layer in semiconducting industry and a dense oxide film of TiO*_X_* will always be formed on the titanium (Ti) metal surface due to the strong affinity with oxygen atoms (O).^[^ ^41^ ^]^ More importantly, when Ti comes into contact with Si substrate, the stable Ti‐O‐Si bonds will be spontaneously formed on surface prior to Ti‐Si bonds due to the inevitable adsorption of O‐containing particles, preventing further reactions of Ti and Si.^[^ ^42^, ^43^ ^]^ Accordingly, Ti layer is promising to be used as a protecting layer between a‐Si:H layer and metal electrodes. In this study, thermally evaporated Ti thin films were introduced into the *n*‐Si/a‐Si:H(*i*)/LiF/Al ESC as a protecting layer, and a preferred structure of *n*‐Si/a‐Si:H(*i*)/LiF/Ti (1.8 nm)/Al contact was chosen by considering both passivation and electron transport properties. This new‐type ESC structure realized an ultralow initial *ρ*_C_ of 7.9 mΩ cm^2^ and an effective surface recombination velocity (*S*_eff_) down to 4.6 cm s^–1^. Most important is that this ESC stack shows extraordinary thermal stability, which can maintain ohmic contact characteristics and outstanding level of passivation on silicon surface after annealing at 200 °C for 20 min in air ambient. By implementing this new‐type ESC, the resulting HSC with a dopant‐free contact for electrons reached an excellent efficiency of 22.12% and maintained >94% of initial efficiency after annealing at 200 °C. Moreover, this robust ESC runs stably and is able to support us complete the fabrication processes with high quality of interdigitated back contacted (IBC) silicon solar cells with dopant‐free contacts for both polarities, i.e., a‐Si:H(*i*)/MoO*_X_*/Ag and a‐Si:H(*i*)/LiF/Ti/Al, receiving a remarkable conversion efficiency of 23.61%. This is the highest efficiency ever reported for silicon dopant‐free solar cells and 1.5% absolutely higher than the best dopant‐free IBC cell. In order to clarify the mechanism behind it, the elemental distribution at the interface of ESC structures by cross‐sectional scanning transmission electron microscopy (STEM) and energy‐dispersive X‐ray spectroscopy (EDX), was reviewed, which reveal that the diffusion of Al into a‐Si:H(*i*) layer is dominant factor for the degradation of passivation and carrier transport properties, while inserting Ti layer can effectively remit this behavior.

## 2 Results and Discussion

### 2.1 Passivation and Contact Properties

**Figure 1:**
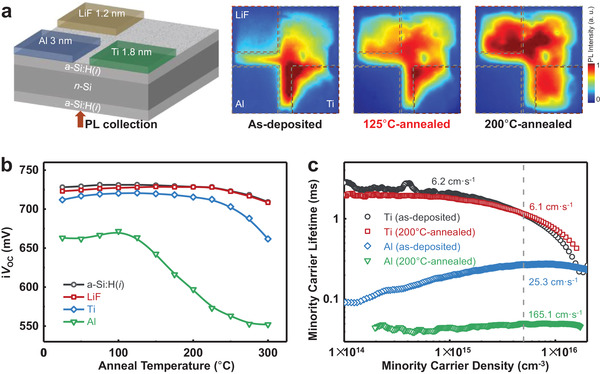
a) The schematic of a a‐Si:H(*i*)/*n*‐Si/a‐Si:H(*i*) sample coated with Ti, Al, and LiF thin films in different region, and the corresponding PL images of the as‐deposited sample, the sample annealed in air ambient at 125 and 200 °C for 5 min, respectively. All PL images were acquired under same parameters. b) Implied *V*_OC_ as a function of sequential 20 min anneals at increased temperature for a‐Si:H(*i*)/*n*‐Si/a‐Si:H(*i*) samples coated with different thin films. c) Injection‐level‐dependent minority carrier lifetimes of as‐prepared Al/a‐Si:H(*i*)/*n*‐Si/a‐Si:H(*i*)/Al, Ti/a‐Si:H(*i*)/*n*‐Si/a‐Si:H(*i*)/Ti samples, and the cases after 200 °C anneal for 20 min. Effective surface recombination velocities are indicated.

Excellent passivation character can be provided by applying the a‐Si:H(*i*) in silicon‐based PV devices, while subsequent processing may degrade the passivation properties. To compare the influence of different coating films on the passivation property of a‐Si:H(*i*), LiF (1.2 nm), Ti (1.8 nm), and Al (3 nm) were deposited on separate regions on the surface of an a‐Si:H(*i*)/*n*‐Si/a‐Si:H(*i*) symmetric sample and the photoluminescence (PL) intensity of each region was tested after successive 20 min anneal with incrementally increasing temperatures up to 200 °C in air ambient. The PL image directly reflects carrier concentration in c‐Si and thus the passivation property of a‐Si:H(*i*) @ coated films. **Figure** **1**a shows the schematic diagram of the testing sample and PL images of the sample for as‐deposited, 125 °C‐ and 200 °C‐annealed cases, with the preparation and testing details shown in the Experimental section. For the as‐prepared sample, the PL signal intensity in the three coated regions were found weaker than the bare a‐Si:H(*i*) region, with the order accordingly signal strength from weak Al‐coated region to strong Ti‐ and LiF‐coated regions. After the sample was annealed, the PL signal intensity was enhanced proportionally with temperatures except for the Al‐coated region. Oppositely, the PL signal from the Al‐coated region decreases as the anneal temperature increases, and ceases changes when the anneal temperature exceeds 125 °C, most probably because the signal is too weak to be detected.

Above PL testing gives an intuitionistic image of passivation quality. In order to implement quantifiable measurement, symmetric structures of a‐Si:H(*i*)/*n*‐Si/a‐Si:H(*i*) wafer coated without/with full‐area Al, Ti, LiF thin layers were fabricated for the examinations with the quasi‐steady‐state photoconductivity technique of a lifetime tester (WCT‐120, Hinton Instruments). The variation of the implied open‐circuit voltage (i*V*_OC_) and the corresponding minority carrier lifetime depending on anneal temperature are shown in Figure 1b and Figure S2 in the Supporting Information, respectively. Similar to the results obtained from PL images, the i*V*_OC_ values of LiF‐ and Ti‐coated samples increased slightly with increasing anneal temperature and persisted up to 200 °C, which is attributed to the repairing effect of post‐annealing on a‐Si:H(*i*) surface defects that are caused by the bombardment of energetic particles during the evaporating process. The i*V*_OC_ of the Al‐coated sample has an initial value of 665 mV, which is ≈60 mV lower than the bare a‐Si:H(*i*) sample (728 mV). Furthermore, it begins to drop significantly when the temperature increases to 125 °C and has fallen below 600 mV at 200 °C. Figure 1c shows the carrier‐injection‐dependent effective carrier lifetime (*τ*_eff_) of Al‐ and Ti‐coated samples without/with anneal at 200 °C, and the corresponding *S*_eff_ are extracted. The *S*_eff_ of Ti‐coated sample is down to ≈6 cm s^–1^, whether it is annealed at 200 °C or not, exhibiting excellent and stable passivation properties of the a‐Si:H(*i*)/Ti stack. In comparison, sample with a‐Si:H(*i*)/Al stack shows poor and unreliable passivation property, with an *S*_eff_ increasing from 25.3 cm s^–1^ (as‐deposited) to 165.1 cm s^–1^ (200 °C‐annealed).

All the evidence shown above suggests that the passivation properties of a‐Si:H(*i*) film dramatically deteriorate when directly contacting it with Al, which may be related to the interdiffusion effect across the interface and even to the occurrence of Al‐induced crystallization on a‐Si:H(*i*). In contrast, coated with Ti and LiF layers do not show the situation mentioned above. Therefore, Ti thin film could be a competitive protecting layer for a‐Si:H(*i*), especially when considering its high compactness (preventing Al diffusion) and good conductivity (maintaining low *ρ*_C_).

**Figure 2:**
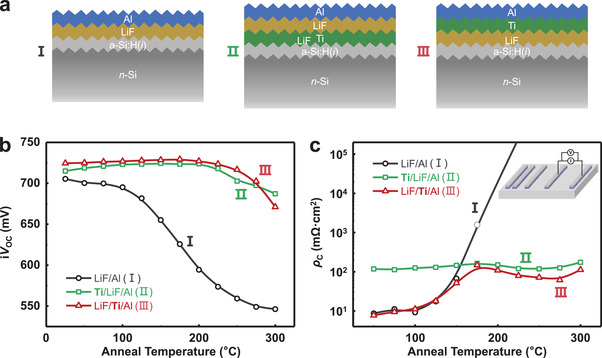
a) Schematic structures of the three ESC stacks and b) their i*V*_OC_ and c) *ρ*_C_ as a function of sequential 20 min anneal at elevated temperatures. Note that the data in (b) are collected from symmetric samples and only one‐side structures are shown in (a). The schematic diagram for extracting *ρ*_C_ is shown as the inset in (c).

In order to investigate the effects of Ti thin‐film as protecting layer for using in electron‐selective passivation contacts, three symmetric samples were fabricated with the following contact stacks: (I) Al/LiF/a‐Si:H(*i*)/*n*‐Si/a‐Si:H(*i*)/LiF/Al; (II) Al/LiF/**Ti**/a‐Si:H(*i*)/*n*‐Si/a‐Si:H(*i*)/**Ti**/LiF/Al; (III) Al/**Ti**/LiF/a‐Si:H(*i*)/*n*‐Si/a‐Si:H(*i*)/LiF/**Ti**/Al, with Ti of 1.8 nm and LiF of 1.2 nm in thickness. Whose one‐sided structures and the variation of corresponding i*V*_OC_ with anneal temperature are shown in **Figure** **2**a,b, respectively. The i*V*_OC_ of the samples with a‐Si:H(*i*)/LiF/Al stack (Stack‐I) initially exceeds 700 mV. However, as in the case of the a‐Si:H(*i*)/Al contact in the previous section, it degrades sharply when anneal temperature exceeds 125 °C and further drops to below 570 mV as anneal temperature reaches 225 °C. In comparison, the i*V*_OC_ values of samples with Ti stacks [a‐Si:H(*i*)/Ti/LiF/Al, Stack‐II and a‐Si:H(*i*)/LiF/Ti/Al, Stack‐III] exceed 715 mV and show approximately an identical trend with increasing anneal temperature, regardless of whether the Ti layer is located above or below the LiF layer. When anneal temperature is below 300 °C, both of them have values greater than 700 mV, and the maximum value reaches 729 mV (Stack‐III, 175 °C). In addition, Figure S3a,b in the Supporting Information show the minority carrier lifetimes of samples with the three stacks after annealed at different temperatures, as well as their injection‐level‐dependent effective lifetime for as‐deposited and 200 °C‐annealed cases, respectively, yielding similar results to the variation of i*V*_OC_. These results demonstrate that relying on the LiF layer itself cannot effectively prevent the destruction of the a‐Si:H(*i*) passivation properties when contacting Al, for both as‐prepared and post‐annealed cases. On the contrary, the insertion of Ti protecting layer can boost passivation quality of the a‐Si:H(*i*) film and remarkably improve thermal stability.

Except for high‐quality passivation that can be characterized by recombination current density (*J*_0_), according to the typical requirements for an optimal ESC contact, high conductivity and electron‐selectivity are also included. This requires ESCs have low and stable *ρ*_C_, enabling electrons be readily transported and collected. Figure 2c shows the variation of *ρ*_C_ with anneal temperatures for the three‐types of ESC stacks extracted by the transfer‐length method (TLM). Both the *n*‐Si/a‐Si:H(*i*)/LiF/Al (black curve) and the *n*‐Si/a‐Si:H(*i*)/LiF/Ti/Al (red curve) possess initial values of *ρ*_C_ less than 10 mΩ cm^2^ and show small changes ranging room temperature to 125 °C, suggesting that the introduction of Ti layer in between LiF and Al does not hinder the carrier transport. With further increasing anneal temperature, *ρ*_C_ of the *n*‐Si/a‐Si:H(*i*)/LiF/Al stack increases sharply and exhibits Schottky contact characteristics with the extracted *ρ*_C_ >1 Ω cm^2^ at 175 °C. This trend from Ohmic to Schottky contact behaviors is undoubtedly a clue of the failure of the insulating LiF as a protecting layer.

For the stack of *n*‐Si/a‐Si:H(*i*)/LiF/Al at low temperature, contact barrier between the a‐Si:H(*i*) and Al can be easily tuned to a very low value due to the interfacial polarity regulation effect by LiF, leading to a quite good Ohmic contact behavior with an ultralow *ρ*_C_. However, along with the increase of anneal temperatures, the thin LiF film cannot play a role of protecting layer and the diffusion of Al across it will cause direct Schottky contact of a‐Si:H(*i*) and Al. Whereas the *n*‐Si/a‐Si:H(*i*)/LiF/Ti/Al (red curve) stack maintains Ohmic contact characteristics continuously from room temperature to 275 °C, although its *ρ*_C_ becomes higher obviously along with temperature. As for the *n*‐Si/a‐Si:H(*i*)/Ti/LiF/Al (green curve), Ohmic contact characteristics with almost unchanged *ρ*_C_ (100–200 mΩ cm^2^) are founded from room temperature to 275 °C. The initial *ρ*_C_ of *n*‐Si/a‐Si:H(*i*)/Ti/LiF/Al is about one order higher than that of *n*‐Si/a‐Si:H(*i*)/LiF/Al and *n*‐Si/a‐Si:H(*i*)/LiF/Ti/Al, showing that the location of the inserted Ti layer in Stack‐II may weaken the effect of interfacial polarity regulation provided by LiF. Therefore, it can be concluded that either inserting Ti protecting layer above or below the LiF can improve the thermal stability of the excellent passivation and contact properties under high‐temperature annealing, while the design of *n*‐Si/a‐Si:H(*i*)/LiF/Ti/Al (Stack‐III) can deliver the best *J*_0_ and *ρ*_C_.

### 2.2 Performance of Dopant‐Free HSCs with a‐Si:H(i)/LiF/Ti/Al Contact

**Figure 3:**
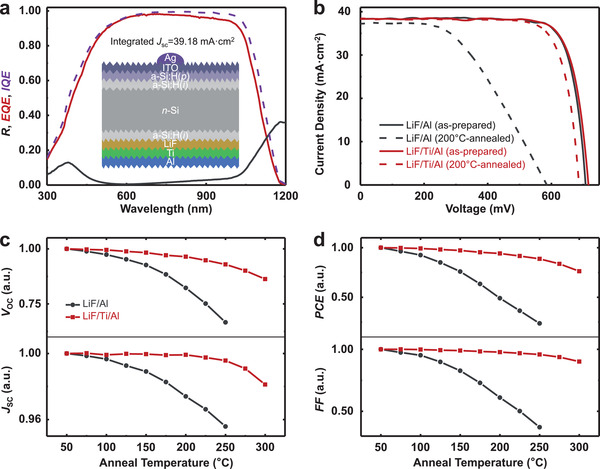
a) EQE, IQE, and *R* of the champion cell with full‐area a‐Si:H(*i*)/LiF/Ti/Al ESC. The inset is a schematic of the cell. b) Light *J*–*V* behavior of as‐prepared and 200 °C‐annealed dopant‐free HSCs with and without Ti protecting layer. Normalized photovoltaic parameters of c) *V*_OC_ and *J*_SC_ and d) PCE and FF after 20 min anneals under different temperatures.

By now, concerning electron‐selective passivating contacts, stabilizing and boosting the passivation and transport properties by introducing Ti protecting layer between LiF and Al layer has been fully demonstrated. To further validate its feasibility on devices, an a‐Si:H(*i*)/LiF/Ti/Al rear ESC in combined with a front a‐Si:H(*i*)/a‐Si:H(*p*)/indium tin oxide (ITO)/Ag conventionally doped hole‐selective contact were adopted to fabricate HSCs [with a full area of 1×1 cm^2^, *n*‐Si substrate], as shown in the inset of **Figure** **3**a. As a control device, a sample with a‐Si:H(*i*)/LiF/Al ESC was also fabricated according to the same specifications. The initial cell with the a‐Si:H(*i*)/LiF/Ti/Al stack possesses a *V*_OC_ of 716.12 mV and a fill factor (FF) of 80.51% and a champion PCE of 22.12% (**Table** **1**). The corresponding external quantum efficiency (EQE), reflection (*R*), and internal quantum efficiency (IQE) obtained by quantum efficiency analysis for this cell are shown in Figure 3a. The *J*_SC_ value extracted from the EQE is 39.18 mA cm^–2^, a little bit higher than the measured *J*_SC_ of 38.36 mA cm^–2^ from the light *J*–*V* curve. As shown in Figure 3b, after annealing for 20 min at 200 °C, although that will not be approached in practical work, the champion cell maintains > 94% of initial efficiency and shows superior thermal stability than the control cell, which maintains only 49% of the initial efficiency. In addition, the measured characteristic parameters of these two types of HSCs are normalized and presented in Figure 3c,d as a function of anneal temperatures in the range of 25–300 °C. It is shown that the degradation of the control cell is mainly caused by *V*_OC_ and FF, which is attributed to the deterioration of its ESC in terms of *S*_eff_ and *ρ*_C_ during annealing, and this problem can be mitigated by the introduction of the Ti protecting layer.

**Table 1:** Light *J*–*V* Parameters of as‐prepared and 200 °C‐annealed dopant‐free HSCs with rear ESC structures of a‐Si:H(i)/LiF/Al and a‐Si:H(i)/LiF/Ti/Al

Samples	Anneal conditions	*V*_OC_ [mV]	*J*_SC_ [mA cm^–2^]	FF [%]	PCE [%]	*J*_0_ [fA cm^–2^]	*ρ*_C_ [mΩ cm^2^]
a‐Si:H(*i*)/LiF/Al	As‐prepared	709.44	38.39	80.60	21.95	23.43	8.69
	200 °C‐annealed	583.52	37.39	49.25	10.74	–	–
a‐Si:H(*i*)/LiF/Ti/Al	As‐prepared	716.12	38.36	80.51	**22.12**	11.72	7.75
	200 °C‐annealed	690.51	38.33	78.68	20.83	10.58	109.03

**Figure 4:**
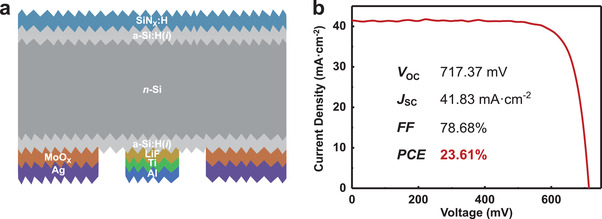
a) Schematic of the dopant‐free IBC HSCs with a‐Si:H(i)/LiF/Ti/Al ESC. b) Light *J*–*V* curve of the champion IBC HSC.

The stable and high‐quality ESC stack enable advanced device structure of IBC configuration combining dopant‐free contacts for both polarities, thereby avoiding any dopants and also boosting light harvesting. A schematic of the IBC cell is shown in **Figure** **4**a, where MoO*_X_* (10 nm)/Ag (300 nm) and LiF (1.2 nm)/Ti (1.8 nm)/Al stacks are exploited as hole‐ and electron‐selective contacts, respectively. As shown in Figure 4b, in combination with an interfacial passivation layer of 6 nm a‐Si:H(*i*) on both sides of an n‐type silicon wafer, it results so far in the highest efficiency of 23.61% with a *V*_OC_ of 717.37 mV, an FF of 78.68%, and a *J*_SC_ of 41.83 mA cm^–2^. Note that the hole‐ and electron‐selective stacks are deposited by only thermal evaporation with the help of two sets of metal shadow masks (Figure S4a–f, Supporting Information), showing huge potential in future in saving equipment investment on expensive plasma‐enhanced‐chemical‐vapor‐deposition (PECVD) for depositing functional layers of a‐Si:H(*p*) and a‐Si:H(*n*) in conventional HIT solar cells. The optimization of the fully dopant‐free IBC device is ongoing work and will be the subject of further publications.

### 2.3 Characterization and Carriers Transport Models

**Figure 5:**
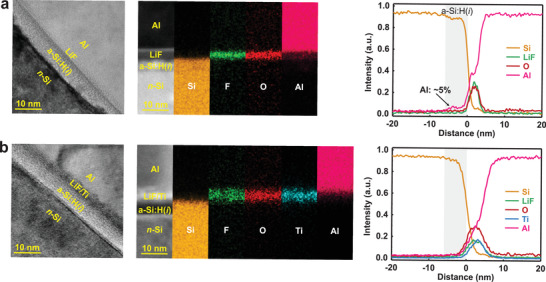
HR‐TEM image, HAADF STEM image, and EDX maps and line profiles of 200 °C‐annealed a) a‐Si:H(*i*)/LiF/Al and b) a‐Si:H(*i*)/LiF/Ti/Al.

**Figure** **5**a shows high‐resolution (HR) TEM and STEM high‐angle annular dark‐field (HAADF) images, and the corresponding EDX maps and line profiles collected from the cross section of the 200 °C‐annealed a‐Si:H(*i*)/LiF/Al structure (Stack‐I). In the HR TEM image, an a‐Si:H(*i*) layer (≈6 nm) and amorphous Al electrode can be clearly distinguished on *n*‐Si substrate, while a LiF layer between them is indistinguishable. The *n*‐Si substrate and Al electrode can be identified in the HAADF STEM image, while both the a‐Si:H(*i*) and the LiF layer are distorted, and there is no defined interface between them. From the EDX maps and profiles, it is known that the concentration of Al is >40% in LiF. More importantly, Al concentration reaches ≈5% at the site of 6 nm below the LiF/a‐Si:H(*i*) interface, in consistent with the thickness of the a‐Si:H(*i*) layer. This indicates that Al has been distributed throughout the a‐Si:H(*i*) layer after annealing treatment at 200 °C, albeit unevenly, forming Al‐Si mixed‐phase or even Al‐doped a‐Si:H(*p*).^[^ ^27^, ^29^ ^]^ These EDX line profiles and maps also show that the O concentration in the LiF layer is ≈20%, probably originating from the oxygen and water molecules adsorbed on a‐Si:H(*i*) surface before coating the following films, which would form stoichiometric oxides with Si and Al.

The counterpart with Ti annealed at 200 °C was performed, as shown in Figure 5b. As a baseline for comparing annealed samples, an as‐prepared a‐Si:H(*i*)/LiF/Ti/Al structure without annealing was also characterized, and the corresponding results are shown in Figure S5 in the Supporting Information. The Ti and LiF layers are found finally form a mixed layer with a thickness of ≈3 nm (≈1.2 nm LiF layer and ≈1.8 nm Ti layer). Although there is no clear boundary between them (Ti and LiF layers), the boundary between them and Al layer is quite clear, differing significantly from the LiF layer in that the Si:H(i)/LiF/Al structure. The formation of mixed LiF/Ti layer was well demonstrated in EDX maps and line profiles in the range of a few nanometers above the a‐Si:H(*i*) surface. In addition, the content of O in LiF/Ti mixed layer is close to 30%, which implies that the Ti layer is in a partially oxidized state at the interface rather than purely metallic state. Although the Ti thin film was prepared by evaporating Ti metal under high vacuum condition (≤5×10^–6^ Torr), partial oxidation is also inevitable due to air residues in the chamber. For the concentration of Al, a significant decrease from the interface between Al and LiF/Ti mixed layer is observed, while it is not found in the a‐Si:H(*i*) layer. This indicates that the Ti layer distributes continuously and covers on the exposed a‐Si:H(*i*) regions, thus avoiding direct contact between Al and a‐Si:H(*i*) layer. It is worth noting that the distribution of interfacial elements, especially for the Al and Ti, does not change markedly for the LiF/Ti/Al structure before (Figure S6a,b, Supporting Information) and after (Figure 5b) 20 min 200 °C‐anneal treatments, indicating that the Ti thin film thoroughly blocked the diffusion of Al into the a‐Si:H(*i*) layer. In addition, the O concentration in the LiF/Ti mixed layer is maintained before and after anneal (Figure S6c, Supporting Information), demonstrating that the 400 nm Al electrode is sufficient to prevent oxygen and other oxidizing species from penetrating the device in the ambient. This both protects the device and eliminates unnecessary interference for sequential research on device degradation under heated conditions.

**Figure 6:**
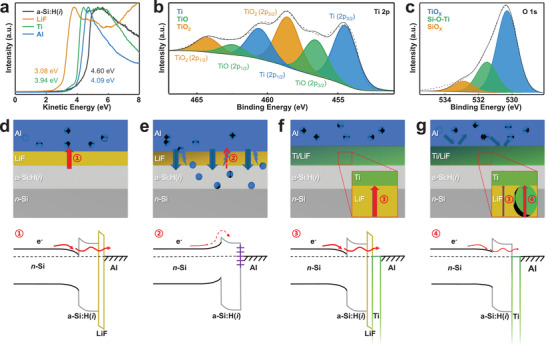
a) Secondary electron cutoff energy (for work‐function) of a‐Si:H(*i*), LiF, Ti, and Al films. XPS core level spectrum of b) Ti 2p and c) O 1s in as‐deposited Ti film. The interfacial composition distribution and the corresponding energy band diagram of d) as‐prepared a‐Si:H(*i*)/LiF/Al, e) 200 °C‐annealed a‐Si:H(*i*)/LiF/Al, f) as‐prepared a‐Si:H(*i*)/LiF/Ti/Al, and g) 200 °C‐annealed a‐Si:H(*i*)/LiF/Ti/Al.

Secondary electron cutoff of the ultraviolet photoelectron spectroscopy (UPS) was used to analyze the photoelectric properties of a‐Si:H(*i*), LiF, Ti, and Al films. As shown in **Figure** **6**a, a low work function value of 3.08 eV can be extracted for the LiF layer compared to the one of Al electrode (4.09 eV), and this low work function value plays a crucial role in eliminating contact barrier and achieving Ohmic characteristics between the Al electrode and *n*‐Si/a‐Si:H(*i*) substrate. In addition, Ti exhibits a slightly lower work function (3.94 eV) than Al electrode, which accounts for the lower initial *ρ*_C_ value obtained when Ti is introduced between LiF and Al in Figure 2c.

The composition of Ti film just deposited on *n*‐Si/a‐Si:H(*i*) substrate is quantified by X‐ray photoelectron spectroscopy (XPS). In order to eliminate disturbance from spontaneous oxidation of the sample exposure to ambient, a much thick Ti film (5 nm) was coated here and the XPS signals were not collected until stripped the outmost ≈3 nm Ti layer with in situ ion beam in XPS instrument. Figure 6b shows that the XPS core‐lever spectrum of Ti 2p consists of Ti, TiO, and TiO_2_ signals with a peak area ratio of 21:12:17, and thus the Ti:O ratio in as‐deposited Ti film is calculated as 13:12. On the other hand, the analysis of the core‐lever spectrum of O 1s in Figure 6c reveals that the O is dominantly located in TiO*_X_* (530.3 eV) and the components of Ti‐O‐Si states are main component at the interface.

Based on the above analysis, it is feasible to establish the interfacial composition distribution and the corresponding energy band diagrams for different ESC stacks, which are favorable to understand the dramatic changes in passivation and transport properties arising from the introduction of Ti layer. Figure 6d,e shows the interfacial composition distribution and energy band diagram of the as‐prepared and 200 °C‐annealed *n*‐Si/a‐Si:H(*i*)/LiF/Al structure, respectively. For the as‐deposited sample, the Al diffusion is not a big issue, and the LiF/Al together can serve as a low work function electrode that enables a favorable downward energy‐band bending (Diagram_1@Path_1) to *n*‐Si/a‐Si:H(*i*) and thus sustains an ultralow *ρ*_C_ for electrons. Meanwhile, the almost undamaged a‐Si:H(*i*) can supply a satisfied passivating level to *n*‐Si substrate. However, the LiF layer cannot block Al diffusion due to its poor protection at elevated temperature. The Al will pass through LiF via a lot of pores and reach the interface of a‐Si:H(*i*)/LiF and then the diffusion depth and concentration of Al in a‐Si:H(*i*) will increase along with increasing anneal temperature. When Al diffuses throughout the a‐Si:H(*i*) layer and is distributed at the *n*‐Si/a‐Si:H(*i*) interface, they act as recombination centers and trap photogenerated electron–hole pairs, leading to severe degradation or even loss of the passivation property of a‐Si:H(*i*) layer on *n*‐Si surface. Simultaneously, *ρ*_C_ of the annealed a‐Si:H(*i*)/LiF/Al stack begins to increase from 100 °C and exhibits Schottky contact characteristic at 175 °C, owing to the Fermi pinning from the direct contact between diffused‐Al and a‐Si:H(*i*) or *n*‐Si, which forms a Schottky barrier (Diagram_2@Path_2) at the interface and hinders electrons from passing through.

As shown in Figure 6f, with the introduction of Ti protecting layer on LiF and also the possible small amount of exposed a‐Si:H(*i*) surface (uncovered portions caused by the ultrathin LiF), the a‐Si:H(i)/LiF/Ti/Al ESC has the best and stable passivation property (Figure 2b). A basic passivation level of this structure stems from a‐Si:H(*i*) layer, similar to the case of a‐Si:H(*i*)/LiF/Al, but the excess in passivation may attribute to additional Ti‐O‐Si bonds. Due to the effective protection of Ti layer, this excellent passivation can be maintained during the 200 °C‐annealing. A low value of initial *ρ*_C_ for *n*‐Si/a‐Si:H(*i*)/LiF/Ti/Al is attributed to the low work function of LiF film for facilitating electron transport (Diagram_3@Path_3), while the Ti layer also plays the role of the metal electrode. However, the *ρ*_C_ value increases to ≈100 mΩ cm^2^ in the subsequent 200 °C‐anneal process, which may originate from the aggregation of LiF that occurs during annealing, resulting in a reduction of its contact area with a‐Si:H(*i*) and an increased portion of contact with Ti with a small downward energy‐band bending (Diagram_3, 4@Path_3, 4), as shown in Figure 6g. Overall, the Ti protecting layer allows the ESC to maintain good passivation and Ohmic characteristics for anneal temperature below 300 °C.

## 3 Conclusions

Dopant‐free carrier selective contacts have not been considered for application to c‐Si solar cell industry until only comparable efficiency and stable performance are achievable. In this study, we developed an approach to stabilize and boost electron‐selective properties of a conventional ESC structure of *n*‐Si/ a‐Si:H(*i*)/LiF/Al by introducing a 1.8 nm Ti protecting layer between LiF/Al. This protecting layer effectively prevents the diffusion of Al into a‐Si:H(*i*) layer and thus keeps a chemically and physically stable interface ranging from room temperature to beyond 200 °C. This preferred structure with *n*‐Si/ a‐Si:H(*i*)/LiF/Ti/Al achieves *ρ*_C_ as low as 7.9 mΩ cm^2^ and *S*_eff_ down to 4.6 cm s^–1^. Based on its excellent passivating level as well as remarkable process stability, we successfully constructed two types of silicon heterojunction solar cells with rear‐sided dopant‐free ESC and interdigitated back dopant‐free contacts (for both polarities), showing particularly high efficiencies of 22.12% and 23.61%, respectively. Even if our record efficiency is 1.5% absolutely higher than the best one reported before, much higher efficiencies can be expected in the near future as the research on this field is just at the beginning.

## 4 Experimental Section

### Measurement of Contact and Passivation Properties

A n‐type (≈1 Ω cm) double‐side texture Czochralsk (Cz) wafers with 140 µm thickness were used. After standard radio corporation of america (RCA) cleaning and diluted hydrofluoric acid (HF) dipping, the wafers were covered symmetrically with 6 nm a‐Si:H(*i*) layer by PECVD. The films of LiF, Ti, Al, or stacks thereof were deposited symmetrically on a‐Si:H(*i*)/c‐Si(*n*) substrates by thermal evaporation according as needed with deposition rates of ≈0.3, 0.3, and 1A s^−1^, respectively. After annealed at different temperatures, the passivation properties of samples were characterized by photoconductance decay (Sinton WCT 120) and Equation (1) calculates the *S*_eff_

(1) $$\begin{array}{c} S_{\mathrm{eff}}=\frac{W}{2}\left(\frac{1}{\tau_{\mathrm{eff}}}-\frac{1}{\tau_{\mathrm{bulk}}}\right) \end{array}$$

where *W* and *τ*_bulk_ are the thickness and bulk lifetime of the c‐Si(*n*) wafer, respectively. Since high‐quality c‐Si(*n*) wafers were used as substrates, the *τ*_bulk_ is assumed to infinity. The *S*_eff_ and saturation current density (*J*_0_) were extracted at a charge carrier density of 5 × 10^15^ cm^–3^. The contact structures with three stacks in Figure 2a were fabricated with the TLM patterns on a‐Si:H(*i*)/c‐Si(*n*) substrates by thermal evaporation through a shadow mask. The *ρ*_C_ was extracted from the dark current‐voltage *I–V* behavior with different pad spacing, which was measured by Keithley 2400 source meter. More details about measurement of contact and passivation properties can be found in the previous papers.^[^ ^44^, ^45^ ^]^ For the sample used for PL measurement, 1.2 nm‐LiF, 1.8 nm‐Ti, and 3 nm‐Al were deposited on different regions of the same a‐Si:H(*i*)/c‐Si(*n*)/a‐Si:H(*i*) substrate backside, and then annealed at different temperatures for PL measurement. It is important to remind that each film was deposited on backside of the substrate to maintaining a uniform concentration of the injected light on the front side. In addition, the thickness of LiF and Ti film is consistent with those applications in the devices, while the thickness of Al is 3 nm to reduce the disturbance of back reflection on the PL signal.

### Devices Fabrication

The same wafers as above were utilized to fabricate both type silicon HSCs. For HSCs with rear‐sided dopant‐free ESC, the front hole‐selective contact consists of a stack of intrinsic (≈6 nm)/boron‐doped a‐Si:H layers (≈10 nm)/ ITO layer (≈80 nm)/Ag grid (≈300 nm). Where a‐Si:H layers, ITO, and Ag were fabricated by PECVD, sputter, and thermal evaporation, respectively, and Ag grid was with a surface fraction of ≈4%. For the rear ESC, an a‐Si:H(*i*) thin layer (≈6 nm) was coated as passivation layer. And then, a full‐area LiF/Al and LiF/Ti/Al stacking structures were prepared sequentially by thermal evaporation. For the dopant‐free IBC HSCs, after standard RCA cleaning and diluted HF dipping, both sides of the wafers were covered with 6 nm a‐Si:H(*i*) by PECVD as interfacial passivation layers. Then, a ≈85 nm SiN*_X_*:H was coated on the front side by PECVD as antireflection layer. After annealing at 200 °C for 30 min in a nitrogen atmosphere, a MoO*_X_* (10 nm)/Ag (300 nm) and a LiF/Ti/Al film were deposited by thermal evaporation with metal shadow masks to act as hole‐selective contact and ESC, respectively. In this dopant‐free IBC HSCs, the width of the hole‐selective contact is 1150 µm while that of the ESC is 350 µm and the center‐to‐center distance (pitch) between them is controlled at 1000 µm while maintaining a fixed space of 250 µm between them.

### Characterization

HR TEM, HAADF‐STEM+EDX was used to characterize the interfacial contact structure of the *n*‐Si/a‐Si:H(*i*)/LiF/Al and *n*‐Si/a‐Si:H(*i*)/LiF/Ti/Al contacts. The composition and work function of films were characterized by XPS and UPS with a Thermo Scientific Escalab 250Xi spectrometer using the Al *K*_*α*_ X‐ray source (*hν* = 1486.6 eV). The device performance of the HSCs was characterized by a solar simulator (Abet Technology, Sun 3000) with a Xe arc lamp under standard test conditions (Air‐mass 1.5 illumination, 1000 W m^–2^, 25 °C) and it utilized a shadow mask with 1×1 cm^2^. An encapsulated standard reference c‐Si solar cell certified by Newport was used to calibrate the illumination intensity. The EQE, IQE, and *R* were measured on the platform of quantum efficiency measurement system (QE‐R3011, Enlitech Co. Ltd.).

The thermal stability of sample performance, including *ρ*_C_, passivation property, PL signal, and device parameters, were annealed on a hotplate for 20 min in air ambient between 50 and 300 °C with a 25 °C interval.

## Conflict of Interest

The authors declare no conflict of interest.

## Supporting information

Supporting Information

## Data Availability

The data that support the findings of this study are available from the corresponding author upon reasonable request.
