# Supplementary material for: Dual Functional Dopant‐Free Contacts with Titanium Protecting Layer: Boosting Stability while Balancing Electron Transport and Recombination Losses
Source: Adv Sci (Weinh). 2022 Jun 15;9(23):2202240. doi: 10.1002/advs.202202240 (PMC9376810; doi:10.1002/advs.202202240)
Supplement: Supplementary file 1 — Supporting Information [file ADVS-9-2202240-s001.pdf]

ADVANCED SCIENCE
Open Access

Supporting Information

for *Adv. Sci.*, DOI 10.1002/advs.202202240

Dual Functional Dopant-Free Contacts with Titanium Protecting Layer: Boosting Stability while Balancing Electron Transport and Recombination Losses

*Zhaolang Liu, Hao Lin, Zilei Wang, Liyan Chen, Taojian Wu, Yicong Pang, Lun Cai, Jian He, Shanglong Peng, Hui Shen and Pingqi Gao**

Supporting Information

Dual Functional Dopant-Free Contacts with Titanium Protecting Layer: Boosting Stability While Balancing Electron Transport and Recombination Losses

*Zhaolang Liu, Hao Lin, Zilei Wang, Liyan Chen, Taojian Wu, Yicong Pang, Lun Cai, Jian He, Shanglong Peng, Hui Shen, and Pingqi Gao**

Dr. Z. Liu, Dr. H. Lin, Dr. Z. Wang, L. Chen, Dr. T. Wu, Y. Pang, Dr. L. Cai, Dr. J. He, Prof. P. Gao
School of Materials, Sun Yat-sen University, Guangzhou 510275, China
E-mail: gaopq3@mail.sysu.edu.cn (P.G.)

Dr. Z. Liu, Prof. S. Peng
National & Local Joint Engineering Laboratory for Optical Conversion Materials and Technology, School of Materials and Energy, Lanzhou University, Lanzhou 730000, China

Prof. H. Shen, Prof. P. Gao
Institute for Solar Energy Systems, Guangdong Provincial Key Laboratory of Photovoltaic Technology, State Key Laboratory of Optoelectronic Materials and Technologies, Sun Yat-sen University, Guangzhou, 510275, China

Prof. P. Gao
Jiangsu Collaborative Innovation Center of Photovoltaic Science and Engineering, Changzhou University, Changzhou, 213164, China

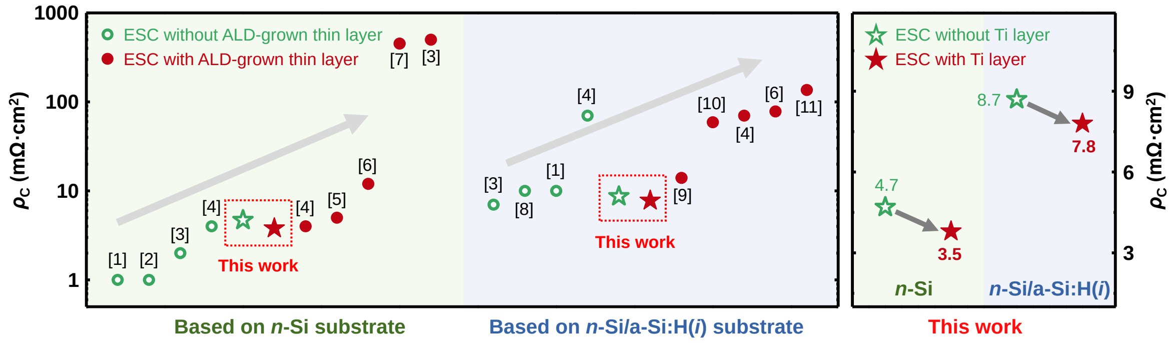

Figure S1. The reported ρ_C of ESCs with/without ALD-grown thin films based on *n*-Si or *n*-Si/a-Si:H(*i*) substrates.[1-11]

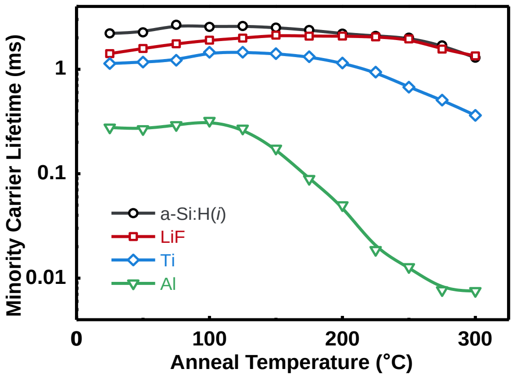

Figure S2. Passivation property of a-Si:H(*i*) layer with different coatings. Minority carrier lifetimes as a function of sequential 20 mins anneals at increasing temperature for samples with different coatings.

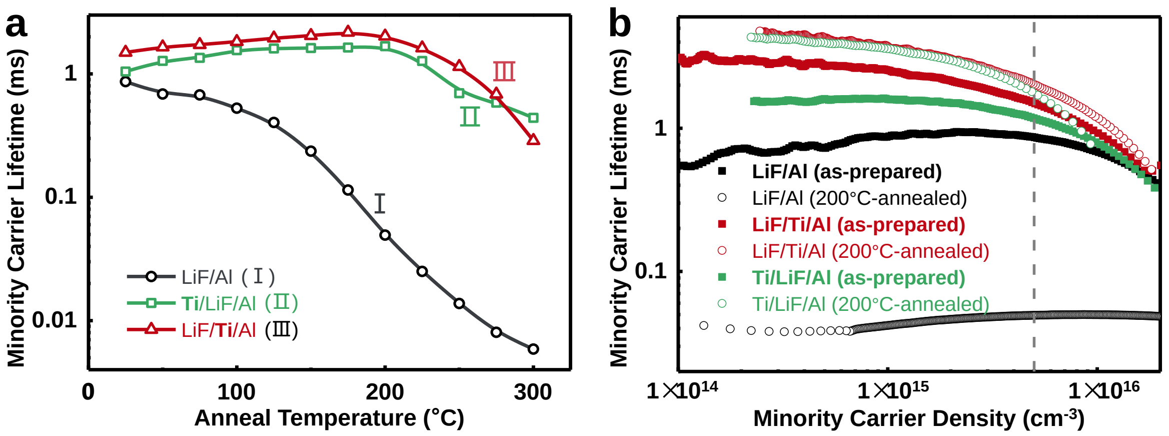

Figure S3. Passivation property of symmetric ESCs on *n*-Si. a) Minority carrier lifetime as a function of sequential 20 mins anneals at increasing temperature for three stacking structures. b) Injection-level-dependent effective lifetimes of as-prepared and 200 °C-annealed of them.

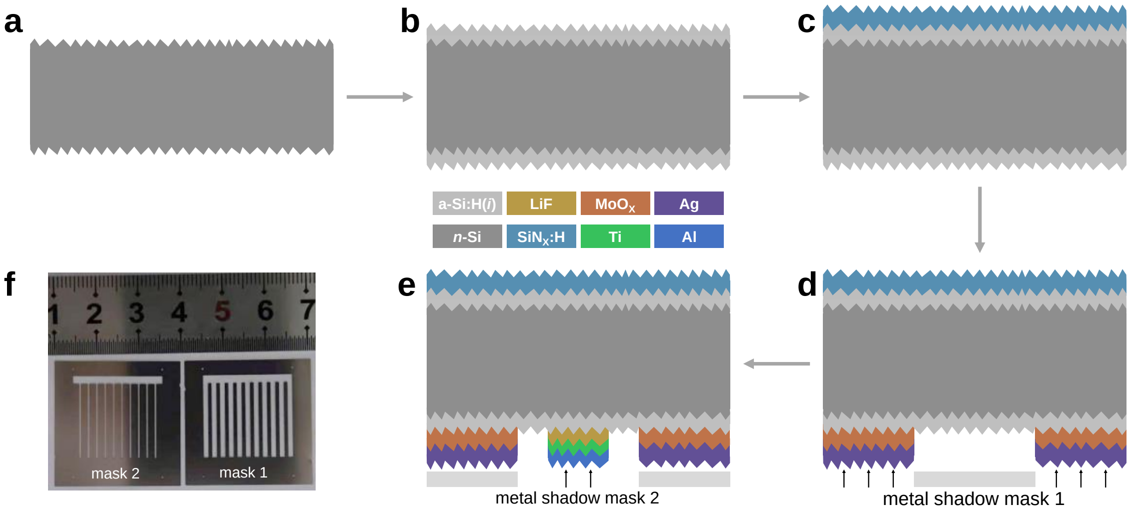

Figure S4. Preparation of the dopant-free IBC HSCs. a)-e) Process flow for the preparation of the dopant-free IBC HSCs. f) Optical image of the two metal shadow masks used.

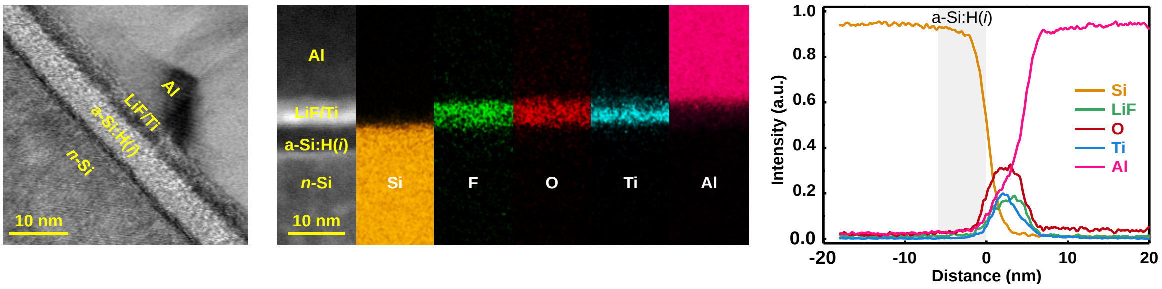

Figure S5. TEM and EDX measurements. HR-TEM image, HAADF STEM image, and EDX maps and line profiles of as-prepared a-Si:H(*i*)/LiF/Ti/Al.

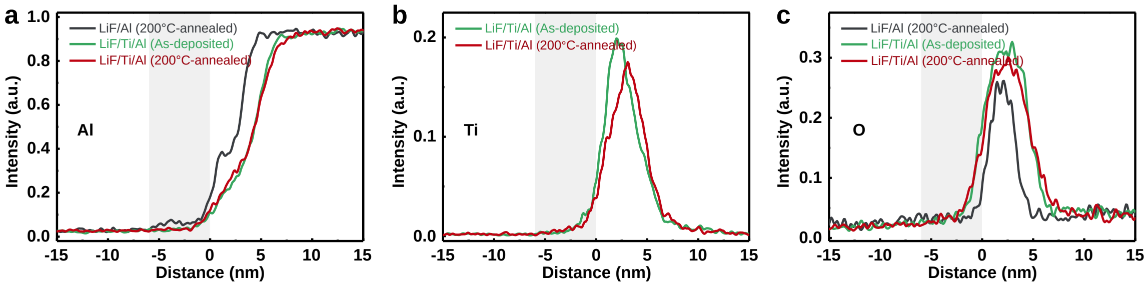

Supplementary Figure S6. Elemental distribution comparison. The distribution of a) Al, b) Ti, and c) O in three ESCs characterized by EDX line profiles.

References

1. W. Wang, J. He, L. Cai, Z. Wang, S. K. Karuturi, P. Gao, W. Shen, *Sol. RRL* **2020**, *4*, 2000569.

2. J. Bullock, P. Zheng, Q. Jeangros, M. Tosun, M. Hettick, C. M. Sutter-Fella, Y. Wan, T. Allen, D. Yan, D. Macdonald, S. De Wolf, A. Hessler-Wyser, A. Cuevas, A. Javey, *Adv. Energy Mater.* **2016**, *6*, 1600241.

3. J. Bullock, M. Hettick, J. Geissbuhler, A. J. Ong, T. Allen, C. M. Sutter-Fella, T. Chen, H. Ota, E. W. Schaler, S. De Wolf, C. Ballif, A. Cuevas, A. Javey, *Nat. Energy* **2016**, *1*, 15031.

4. J. Bullock, Y. Wan, Z. Xu, S. Essig, M. Hettick, H. Wang, W. Ji, M. Boccard, A. Cuevas, C. Ballif, A. Javey, *ACS Energy Lett.* **2018**, *3*, 508.

5. T. G. Allen, J. Bullock, Q. Jeangros, C. Samundsett, Y. Wan, J. Cui, A. Hessler-Wyser, S. De Wolf, A. Javey, A. Cuevas, *Adv. Energy Mater.* **2017**, *7*, 1602606.

6. X. Yang, Y. Lin, J. Liu, W. Liu, Q. Bi, X. Song, J. Kang, F. Xu, L. Xu, M. N. Hedhili, D. Baran, X. Zhang, T. D. Anthopoulos, S. De Wolf, *Adv. Energy Mater.* **2020**, *32*, 2002608.

7. X. Yang, E. Aydin, H. Xu, J. Kang, M. Hedhili, W. Liu, Y. Wan, J. Peng, C. Samundsett, A. Cuevas, S. De Wolf, *Adv. Energy Mater.* **2018**, *8*, 1800608.

8. J. Wang, H. Lin, Z. Wang, W. Shen, J. Ye, P. Gao, *Nano Energy* **2019**, *66*, 104116.

9. W. Lin, M. Boccard, S. Zhong, V. Paratte, Q. Jeangros, L. Antognini, J. Dreon, J. Cattin, J. Thomet, Z. Liu, Z. Chen, Z. Liang, P. Gao, H. Shen, C. Ballif, *ACS Appl. Nano Mater.* **2020**, *3*, 11391.

10. J. Cho, M. Debucquoy, M. R. Payo, S. Malik, M. Filipič, H. S. Radhakrishnan, T. Bearda, I. Gordon, J. Szlufcik, J. Poortmans, *Energy Procedia.* **2017**, *124*, 842.

11. S. Zhong, J. Dreon, Q. Jeangros, E. Aydin, S. De Wolf, F. Fu, M. Boccard, C. Ballif, *Adv. Funct. Mater.* **2019**, *30*, 1907840.
